# Radiation Quality-Dependent Progressive Increase in Oxidative DNA Damage and Intestinal Tumorigenesis in *Apc*^1638N/+^ Mice

**DOI:** 10.3390/curroncol32070382

**Published:** 2025-07-01

**Authors:** Kamendra Kumar, Santosh Kumar, Jerry Angdisen, Kamal Datta, Albert J. Fornace, Shubhankar Suman

**Affiliations:** 1 Department of Oncology, Lombardi Comprehensive Cancer Center, Georgetown University Medical Center, Washington, DC 20057, USA; kk1264@georgetown.edu (K.K.);; 2 Department of Biochemistry and Molecular & Cellular Biology, Georgetown University Medical Center, Washington, DC 20057, USA

**Keywords:** ionizing radiation, heavy-ion radiation, 8-OxodG, cancer, biomarker

## Abstract

Exposure to high-linear energy transfer (LET) heavy ions, such as ^28^Si, poses a significant cancer risk for astronauts. While previous studies have linked high-LET radiation exposure to persistent oxidative stress and dysregulated stress responses in intestinal crypt cells with an increased risk of tumorigenesis, the relationship between IR-induced oxidative DNA damage and intestinal cancer risk remains incompletely understood. Here, we investigated the time-dependent effects of ^28^Si-ion radiation on intestinal tumorigenesis and oxidative DNA damage in *Apc*^1638N/+^ mice, a model for human intestinal cancer predisposition. Male *Apc*^1638N/+^ mice were exposed to 10 cGy of either γ-rays (low-LET) or ^28^Si-ions (high-LET), and intestinal tumor burden was assessed at 60 and 150 days post-irradiation. While both radiation groups showed modest, non-significant tumor increases at 60 days, ^28^Si-irradiated mice exhibited an approximately 2.5-fold increase in tumor incidence by 150 days, with a higher incidence of invasive carcinomas compared to γ and sham groups. Serum 8-OxodG levels, a marker of systemic oxidative stress, were significantly elevated in the ^28^Si-ion group, correlating with increased intestinal 8-OxodG staining. Additionally, assessment of the proliferation marker Cyclin D1 and metaplasia marker Guanylyl Cyclase C (GUCY2C) also revealed significant crypt cell hyperproliferation accompanied by increased metaplasia in ^28^Si-exposed mouse intestines. Positive correlations between serum 8-OxodG and tumor-associated endpoints provide compelling evidence that exposure to ^28^Si-ions induces progressive intestinal tumorigenesis through sustained oxidative DNA damage, crypt cell hyperproliferation, and metaplastic transformation. This study provides evidence in support of the radiation quality-dependent progressive increase in systemic and intestinal levels of 8-OxodG during intestinal carcinogenesis. Moreover, the progressive increase in oxidative DNA damage and simultaneous increase in oncogenic events after ^28^Si exposure also suggest that non-targeted effects might be a significant player in space radiation-induced intestinal cancer development. The correlation between serum 8-OxodG and oncogenic endpoints supports its potential utility as a predictive biomarker of high-LET IR-induced intestinal carcinogenesis, with implications for astronaut health risk monitoring during long-duration space missions.

## Simple Summary

Astronauts on long-term space missions are exposed to ionizing radiation from heavy ions like ^28^Si, which increases cancer risk. Using a mouse model that mimics human intestinal cancer, we compared the effects of ^28^Si radiation to γ-rays. We found that, compared to γ-rays, ^28^Si caused a significantly higher number of intestinal tumors over time, closely linked to persistent oxidative DNA damage. This damage was marked by increased levels of 8-OxodG—a key indicator of DNA injury—in both blood and gut tissue, along with abnormal cell growth in the intestinal tract. These results suggest that 8-OxodG may serve as a useful biomarker to monitor cancer risk in space. This study also highlights potential treatments, such as senotherapeutic drugs, that may help lower this risk by reducing DNA damage and enhancing repair. These findings could improve cancer risk monitoring for astronauts and inform safer radiation treatments on Earth.

## 1. Introduction

Exposure to ionizing radiation (IR) in space is recognized as a major health risk for astronauts, including an elevated risk of cancer during and after long-duration deep space missions [1,2]. Unlike terrestrial exposures, which primarily involve low-linear energy transfer (LET) radiation such as γ-rays, deep space IR exposure is dominated by high-LET heavy ions, such as ^28^Si-ions [3,4]. Radiation quality describes the physical characteristics of radiation, including energy (E), LET, and ionization density. LET refers to the average amount of energy deposited per unit path length of tissue traversal, with higher LET associated with denser ionization tracks [5,6]. High-LET radiation exhibits a greater relative biological effectiveness (RBE) relative to low-LET radiation, meaning it induces a more severe biological effect per unit dose [7,8,9,10,11]. In addition to direct DNA damage, at lower sublethal doses, high-LET IR is estimated to elicit significant non-targeted effects (NTEs) that include persistent oxidative stress-induced DNA damage response (DDR) in cells not directly traversed by radiation tracks, that could amplify tissue-wide damage [12,13,14]. The elevated RBE for carcinogenic risk is largely attributed to the greater ability of low-dose high-LET radiation to induce NTEs compared to equivalent doses of low-LET radiation [15,16,17]. However, the association between persistent oxidative stress, i.e., a significant contributor to the NTE, and oncogenic events remains poorly understood, particularly in the context of radiation quality.

The intestinal epithelium is among the most sensitive tissues to IR-induced damage [18,19]. In space-relevant exposure scenarios, such as low-dose sublethal IR exposure, intestinal epithelial cells have been shown to exhibit concurrent accumulation of persistent oxidative stress, ongoing DNA damage accumulations, and activation of pro-inflammatory oncogenic signaling [20,21]. Following sublethal IR exposure, most intestinal epithelial cells continue to divide; however, a subset with unrepaired or misrepaired DNA may undergo senescence (permanent cell cycle arrest) or aberrant proliferation and homeostasis [21]. Importantly, some IR-induced senescent cells can develop a senescence-associated secretory phenotype (SASP), characterized by the secretion of pro-inflammatory and oncogenic factors that can disrupt the tissue microenvironment and promote tumor development [21]. High-LET IR, in particular, induces greater chronic accumulation of oxidative DNA lesions, notably 8-oxo-7,8-dihydro-2′-deoxyguanosine (8-OxodG), compared to low-LET IR [22]. 8-OxodG is a mutagenic lesion known to mispair with adenine during DNA replication, resulting in G:C to T:A transversions that are strongly associated with tumor initiation and progression [23,24]. Proliferation of cells with sublethal but elevated 8-OxodG levels plays important role in tumor development as it preserves viability while allowing the accumulation of mutations and epigenetic alterations [25,26,27]. These molecular alterations in intestinal epithelial stem cells are known to develop into metaplasia marked by higher expression of Guanylyl cyclase C (GUCY2C) [28,29,30], which is a premalignant condition that increases the risk of progression to dysplasia, adenoma, and adenocarcinoma [31,32].

In this study, we utilized the well-established *Apc*^1638N/+^ mouse model of human colorectal cancer (CRC) [32,33] to investigate the correlation between systemic 8-OxodG levels and heavy-ion-induced intestinal cell phenotypes associated with increased risk of intestinal tumorigenesis. The progression of intestinal tumors in the *Apc*^1638N/+^ model closely recapitulates the sequence of events observed in human CRC, progressing from normal epithelium → metaplasia → dysplasia → adenoma (polyp) → adenocarcinoma [34]. Male *Apc*^1638N/+^ mice were exposed to a space-relevant dose (10 cGy) of ^28^Si-ions, with a parallel cohort receiving the same dose of γ-rays for comparison. To elucidate the relationship between intestinal tumor development and systemic oxidative DNA damage, we assessed markers of oxidative stress-associated DNA damage (8-OxodG) [27,28], cell proliferation (Cyclin D1) [35], and metaplasia (GUCY2C)] [28,29,30] in intestinal tissues at 60 and 150 days post-exposure. Serum 8-OxodG levels were also quantified to explore its potential as a systemic biomarker of IR-induced intestinal cancer risk.

## 2. Materials and Methods

### 2.1. Animal Breeding, Housing, and Irradiations

Male *Apc*^1638N/+^ mice on a C57BL/6 background were bred in-house at Georgetown University (GU) by crossing with C57BL/6J females (JAX strain #: 000664, The Jackson Laboratory, Bar Harbor, ME, USA). Offspring were genotyped as previously described [36], and male *Apc*^1638N/+^ mice were used. Animals were group-housed (five per cage) under specific pathogen-free conditions in individually ventilated cages, maintained at 22 °C with 50% relative humidity, a 12:12 h light/dark cycle, and provided unrestricted access to standard rodent chow and filtered water.

At 7–9 weeks of age, animals were randomly assigned to either control (sham) or irradiation groups and exposed to γ-rays or ^28^Si-ions. Irradiation with ^28^Si (10 cGy, 300 MeV/n, 69 keV/μm) ions was performed at the NASA Space Radiation Laboratory (NSRL) located at Brookhaven National Laboratory (BNL), Upton, NY, USA. Separately, a cohort of mice was exposed to 10 cGy of γ-rays using a ^137^Cs irradiator at GU’s radiation facility. All irradiated and control animals were returned to GU via approved same-day animal transport and housed under identical environmental conditions for the remainder of the study. All animals underwent identical handling, restraint, and transport procedures without receiving radiation. Post-irradiation, mice were monitored twice daily for clinical signs of discomfort or illness, including decreased activity, hunched posture, diarrhea, or weight loss exceeding 15% relative to cage mates. Animals showing persistent or severe distress were humanely euthanized using CO_2_ asphyxiation within 4–6 h of detection and excluded from final analyses. All experimental procedures were performed in accordance with protocols approved by the Institutional Animal Care and Use Committees (IACUC) at GU (#2016-1129, valid through 10 December 2027) and BNL (#345, valid through 6 February 2024).

### 2.2. Euthanasia, Biospecimen Collection, and Tumorigenesis Analysis

**Figure 1:**
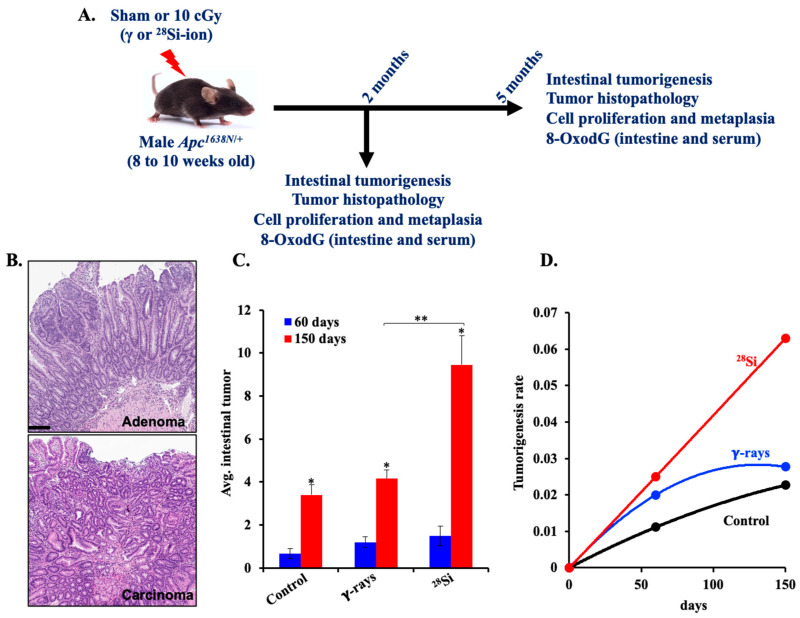
Effect of radiation quality on intestinal tumorigenesis in male *Apc*^1638N/+^ mice. (**A**) Schematic summary of the experimental plan and sample collection. (**B**) Representative photomicrographs of hematoxylin and eosin-stained intestinal adenoma and carcinoma. Scale bar: 200 μm. (**C**) Quantitation of tumor numbers in the small intestine of *Apc*^1638N/+^ mice [at 60 days (control *n* = 12, γ-rays *n* = 10, and ^28^Si-ion *n* = 10) and 150 days (*n* = 20 mice/group)]. (**D**) Tumorigenesis rate. Bars indicate the mean ± SEM and statistically significant change is marked by * (relative to 60-day time point of same group) and ** (relative to γ rays at 150-day time point).

A schematic summary of the experimental plan, biospecimen collection, and subsequent analysis is depicted in Figure 1A. All experimental mice were humanely euthanized using a carbon dioxide (CO_2_) chamber with a regulated flow rate set between 30 and 60% of the cage volume per minute, in accordance with institutional animal care guidelines. Whole blood was collected immediately via cardiac puncture and transferred into serum separator tubes (BD Microtainer^®^ Ref. No. 365967, Becton, Dickinson and Company, Franklin Lakes, NJ, USA). Samples were processed promptly following the manufacturer’s protocol to isolate serum, which was then aliquoted, flash-frozen in liquid nitrogen, and stored at −80 °C until further analysis.

Following euthanasia, intestinal tissues were carefully dissected, rinsed in phosphate-buffered saline to remove luminal contents, and longitudinally opened. Gross tumors in the intestinal tract were scored under a dissecting microscope (Leica MZ6) by multiple independent observers blinded to treatment groups and the average number of intestinal tumors in each experimental group was obtained. Tumorigenesis data were presented as mean ± SEM, and intergroup comparisons were made to assess temporal progression and radiation quality-dependent effects on tumor development. To assess the tumorigenic potential of different radiation exposures, mean value of intestinal tumors was evaluated in mice exposed to sham (control), γ-rays, or ^28^Si-ion radiation at 60 days [control (*n* = 12), γ-rays (*n* = 10), and ^28^Si-ion (*n* = 10)] and 150 days [*n* = 20 mice/group]. Tumor counts were recorded at two defined time points: 60 and 150 days post-IR and tumorigenesis rates [tumorigenesis per unit time (days)] were calculated independently for each group. Representative segments of both normal and tumor-bearing tissue were fixed in 10% neutral-buffered formalin, followed by paraffin embedding. Formalin-fixed paraffin-embedded (FFPE) tissues were sectioned at 4–6 µm thickness using a microtome for downstream histological and immunohistochemical (IHC) analysis. Hematoxylin and eosin (H&E)-stained sections were scored as either adenoma (premalignant) or carcinoma (malignant) based on assessments of crypt architecture, cellular atypia, differentiation, crypt/villus alterations, and muscularis mucosa invasion (Figure 1B).

### 2.3. Serum 8-OxodG Quantitation by ELISA

Quantification of 8-OxodG levels in mouse serum (*n* = 6/group) was performed using a commercially available kit (4370-096-K, Trevigen, Gaithersburg, MD, USA). Serum samples were diluted 1:10 in the assay diluent buffer and loaded in duplicate into a 96-well ELISA plate. Samples from all experimental groups at 60- and 150-day time points were analyzed simultaneously to minimize inter-assay variability. Absorbance was measured at 450 nm using a microplate reader, and 8-OxodG concentrations were calculated as per the manufacturer’s protocol.

### 2.4. Immunohistochemical Staining of Intestinal Tissue Section

Immunohistochemistry (IHC) was performed to assess the levels of 8-OxodG, Cyclin D1, and GUCY2C in formalin-fixed, paraffin-embedded (FFPE) intestinal tissue sections (*n* = 5). Sections (5 µm) were deparaffinized, rehydrated through graded alcohols, and subjected to heat-induced antigen retrieval using citrate buffer (pH 6.0; Electron Microscopy Sciences, Hatfield, PA, USA). IHC was conducted using the Mouse and Rabbit Specific HRP/DAB Detection Kit (ab236466, Abcam, Boston, MA, USA). Tissue sections were incubated overnight at 4 °C in a humidified chamber with the following primary antibodies: anti-8-OxodG (4354-MC-050, 1:200; Trevigen, Gaithersburg, MD, USA), anti-cyclin D1 (MA5-14512, 1:100; Invitrogen, Waltham, MA, USA), and anti- GUCY2C (ab213430, 1:100; Abcam, Boston, MA, USA). After three washes with PBS, sections were incubated with an HRP-conjugated secondary polymer provided in the detection kit, followed by chromogenic development using DAB substrate, as per the manufacturer’s protocol. Nuclear counterstaining was performed using hematoxylin (Electron Microscopy Sciences), after which sections were dehydrated, cleared, and mounted using Permount medium (SP15-100, Fisher Chemical, Frederick, MD, USA).

### 2.5. Imaging and Data Analysis

Stained slides were imaged using the Aperio whole slide scanner (Leica Biosystems) at 20× magnification, and representative high-power fields (HPFs) were selected for quantitative analysis. DAB-positive cells were quantified using Fiji (ImageJ2) software v2.9.0/1.53t (NIH) by applying a color threshold specific to the DAB chromogen (brown) using the H-DAB deconvolution plugin. Threshold parameters were optimized and held constant across all samples to ensure consistency. Positive cells within each HPF were counted, and numbers of DAB-positive cells per HPF from control and IR-exposed tissue sections were obtained. At least 5 HPFs per sample (*n* = 5/group) were analyzed, and the mean count was calculated. Quantification data were normalized to control samples and represented as fold change over control.

### 2.6. Statistical Analysis

Statistical analyses were performed using GraphPad Prism software v6.0a for Mac (La Jolla, CA, USA). To assess data variability and distribution in the quantitative analysis of intestinal tumors, a non-parametric test for equality of variance was applied. Group comparisons were conducted using one-way ANOVA followed by Dunn’s multiple comparisons test to determine statistically significant differences (*p* < 0.05) between control and IR-exposed groups. For ELISA and IHC quantifications, a two-tailed paired Student’s t-test was employed to evaluate significance (*p* < 0.05). Data are presented as bar graphs displaying group means, with error bars representing the standard error of the mean (SEM). Differences were considered statistically significant when *p* values were less than 0.05.

To explore potential trends in biological responses following IR exposure, we evaluated the correlation between post-exposure time and the ^28^Si-to-γ response ratio across four endpoints: (1) tumorigenesis, (2) cell proliferation, (3) metaplasia, and (4) serum 8-OxodG levels. The Pearson correlation coefficient (r) was calculated to assess the linear relationship between post-exposure duration and the ^28^Si-to-γ response ratio. Given that only two data points were available per group, r values were either +1.0 or −1.0, indicating a perfect positive or negative linear trend, respectively.

## 3. Results

### 3.1. Radiation Quality-Dependent Progression of Intestinal Tumorigenesis in Apc^1638N/+^ Mice

Intestinal tumor and cancer incidence was evaluated in male *Apc*^1638N/+^ mice, at 60 and 150 days following exposure to either low-linear energy transfer (LET) γ-rays or high-LET ^28^Si-ion radiation. Quantitative analysis of intestinal tumor burden showed a modest, statistically insignificant increase in tumor incidence in both γ- and ^28^Si-ion-exposed mice at 60 days post-irradiation relative to controls (Figure 1C).

By contrast, a pronounced, time-dependent elevation in tumor number, i.e., ~2.5-fold higher, was observed in the ^28^Si-ion-irradiated group compared to both γ-irradiated and sham controls at 150 days post-exposure (Figure 1C). Furthermore, a notable rise in the rate of tumorigenesis was observed in the ^28^Si-ion group over time, whereas γ-irradiated mice exhibited a plateau effect by day 150 (Figure 1D). Histopathological analysis of intestinal tumor sections stained with hematoxylin and eosin revealed differences in tumor grade and progression across experimental groups. At 60 days post-IR, no carcinomas were observed in any group. However, by 150 days post-irradiation, carcinoma was detected in 26.9% of tumors (14 out of 52) in the ^28^Si-ion-irradiated group, compared to 5.1% (2 out of 39) in the γ-irradiated group and 3.2% (1 out of 31) in sham controls. These findings suggest that high-LET ^28^Si-ion radiation induces persistent, progressive adverse effects on intestinal tumorigenesis, highlighting a potential higher long-term cancer risk.

### 3.2. ^28^Si-Ion Exposure Induced Progressive Accumulations of 8-OxodG in Apc^1638N/+^ Mouse Serum

**Figure 2:**
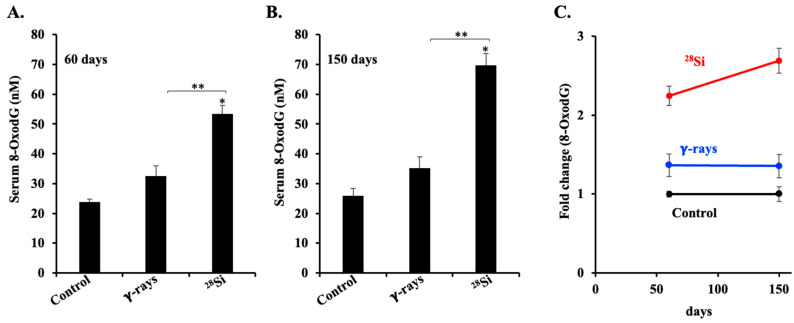
Effect of radiation quality on serum 8-OxodG in male *Apc*^1638N/+^ mice. (**A**) Serum 8-OxodG level in each group at 60 days post-IR. (**B**) Serum 8-OxodG level in each group at 150 days post-IR (*n* = 6/group). (**C**) Fold change of 8-OxodG shows more pronounced effects of ^28^Si-ion radiation at both 60 and 150 days post-IR. Bars indicate the mean ± SEM and statistically significant change is marked by * (relative to sham control) and ** (relative to γ rays).

Oxidative DNA damage is a critical contributor to mutagenesis and tumorigenesis [37,38]. Serum 8-OxodG levels in mice exposed to ^28^Si-ion radiation at 60 days post-irradiation exhibited a significant elevation in serum 8-OxodG compared to both sham controls and γ-irradiated mice (Figure 2A). This effect was further amplified at the 150-day post-irradiation time point, where ^28^Si-ion-exposed mice showed an appreciably higher increase in 8-OxodG levels relative to both control and γ-irradiated groups (Figure 2B). Fold change analysis confirmed a time-dependent increase in serum 8-OxodG in the ^28^Si-ion group, indicating sustained progressive increase in systemic oxidative stress over time. In contrast, no noticeable time-dependent change was observed in the γ-irradiated group (Figure 2C). These findings suggest that ^28^Si-ion radiation induces prolonged oxidative DNA damage, potentially contributing to delayed carcinogenic effects.

### 3.3. ^28^Si-Ion Exposure Induced Progressive Accumulations of 8-OxodG in Apc^1638N/+^ Mouse Intestine

**Figure 3:**
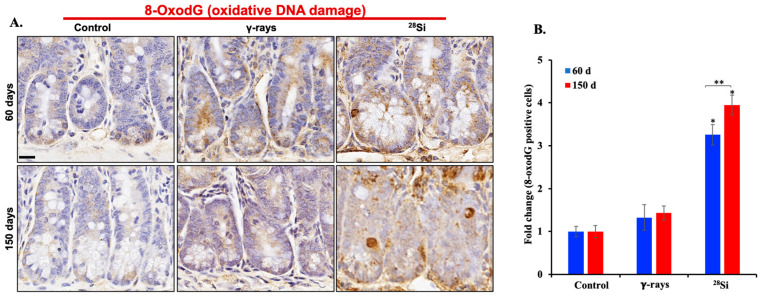
Oxidative DNA damage in intestinal epithelium. (**A**) Representative image of IHC-stained intestinal tissue sections depicting 8-OxodG accumulation at 60- and 150-day time points in sham, and irradiated groups. (**B**) Quantification of 8-OxodG in intestinal crypt region at 60- and 150-day time points in sham, and irradiated groups (*n* = 5/group). Scale bar: 20 μm. Bars indicate the mean ± SEM and statistically significant change is marked by * (relative to sham control) and ** (relative to 60-day time point of the same group).

To evaluate tissue-specific oxidative DNA damage, we performed immunohistochemical staining for 8-OxodG in intestinal sections of *Apc*^1638N/+^ mice. A noticeable increase in 8-oxodG-positive nuclei was observed within the intestinal crypts of ^28^Si-ion-irradiated mice compared to both sham controls and γ-irradiated mice at 60 and 150 days post-exposure (Figure 3A). Quantitative analysis confirmed significantly higher 8-OxodG expression in the intestinal tissue of ^28^Si-exposed mice at both time points relative to controls and γ-irradiated groups (Figure 3B). Additionally, there was a significant time-dependent increase in serum 8-OxodG in the ^28^Si-ion group (Figure 2B). In contrast, no noticeable time-dependent change was observed in the γ-irradiated group. While γ-radiation also induced 8-OxodG expression slightly above control levels, the magnitude of increase was ~2-fold greater following ^28^Si exposure.

### 3.4. ^28^Si-Ion Exposure Induced Cell Proliferation and Metaplasia in Apc^1638N/+^ Mouse Intestine

**Figure 4:**
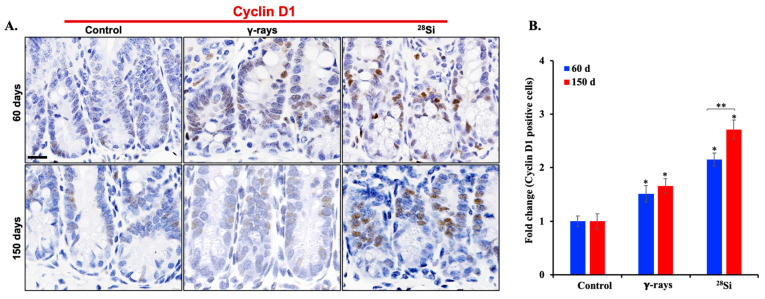
Increased expression of cell proliferation marker cyclin D1 in intestinal epithelial cells after ^28^Si-ion irradiation. (**A**) Representative image of IHC-stained intestinal tissue sections showing increased number of cyclin D1-positive cells at 60 and 150 days post ^28^Si-ion radiation. (**B**) Quantification of cyclin D1-positive cells in intestinal crypt shows significantly increased number of proliferating cells at 150 days compared to 60 days in ^28^Si-ion-irradiated samples (*n* = 5/group). Scale bar: 20 μm. Bars indicate the mean ± SEM and statistically significant change is marked by * (relative to sham control) and ** (relative to 60-day time point of the same group).

To assess the impact of ^28^Si-ion radiation on intestinal epithelial cell dynamics, we investigated the expression of markers associated with cell proliferation and metaplasia, i.e., cyclin D1 and GUCY2C. Immunohistochemical analysis of intestinal sections revealed a marked increase in cyclin D1-positive nuclei within the crypt regions of ^28^Si-irradiated *Apc*^1638N/+^ mice compared to both sham controls and γ-irradiated groups at 60 and 150 days post-exposure (Figure 4A).

**Figure 5:**
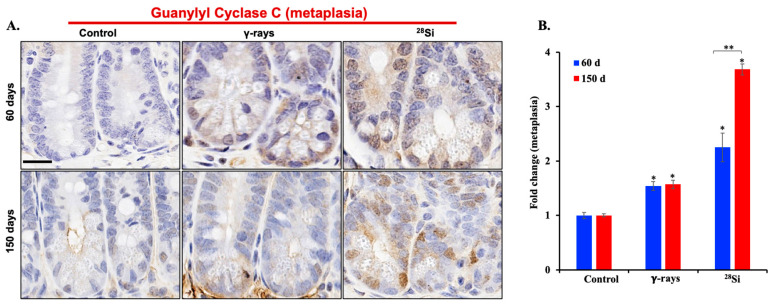
Radiation quality-dependent increase in the number of metaplastic cells in the male *Apc*^1638N/+^ mouse intestine. (**A**) Representative image of IHC-stained intestinal tissue sections showing increased number of GCC positive cells (a marker of intestinal metaplasia) at 60 and 150 days post ^28^Si-ion radiation. (**B**) Quantification of GCC expression (*n* = 5/group). Scale bar: 20 μm. Bars indicate the mean ± SEM and statistically significant change is marked by * (relative to sham control) and ** (relative to 60-day time point of the same group).

Quantitative evaluation confirmed significantly elevated cyclin D1 expression in the ^28^Si-exposed group at the 150-day time point, suggesting sustained and enhanced proliferative activity (Figure 4B). Immunohistochemical staining for GUCY2C demonstrated increased metaplasia in the crypts of ^28^Si-irradiated mice relative to sham and γ-exposed animals at both 60 and 150 days post-exposure (Figure 5A). Quantitative analysis supported a significant elevation in GUCY2C-positive cells following ^28^Si exposure, indicating IR-induced metaplastic transformation in the intestinal epithelium (Figure 5B). Together, these results highlight the ability of ^28^Si-ion radiation to induce persistent proliferative and metaplastic changes in the intestinal crypts, which may contribute to the elevated intestinal tumorigenesis observed in this model.

### 3.5. Systemic Increase in 8-OxodG Positively Correlates with Tumorigenesis-Associated Endpoints

**Figure 6:**
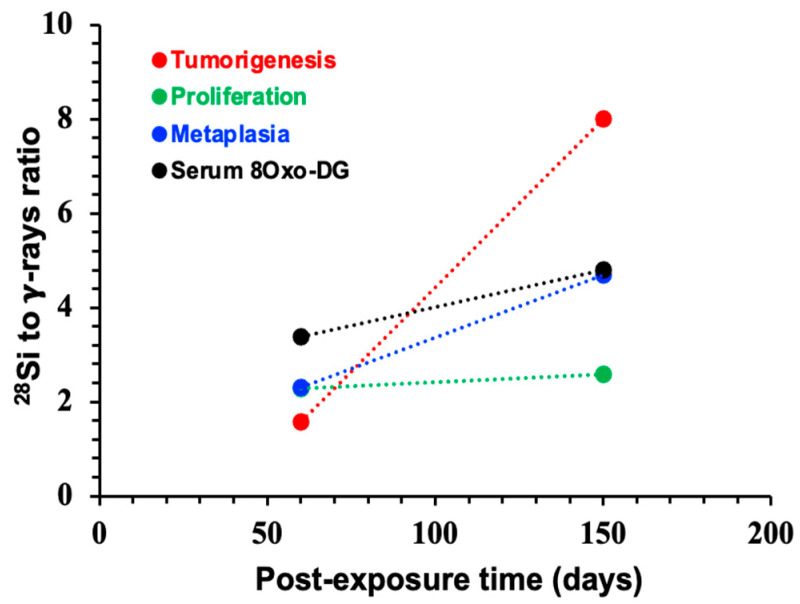
Time-dependent increase in serum levels of 8-OxodG shows positive correlation with tumor-promoting endpoints (i.e., cell proliferation, metaplasia, and tumor number) following ^28^Si-ion exposure. We analyzed the correlation between post-exposure time (60 and 150 days) and the ^28^Si-to-γ response ratio across all endpoints. Pearson correlation coefficients (r) were calculated to quantify linear relationships; due to the use of only two time points, r values were either +1.0 or −1.0, indicating positive or negative correlations, respectively. Notably, all four endpoints, including serum 8-OxodG, exhibited a positive correlation (r = +1.0) with increasing post-exposure time in the ^28^Si-to-γ response ratio, suggesting that the biological impact of ^28^Si-ion radiation intensifies over time, relative to low-LET γ-rays.

To investigate the relevance of oxidative stress-associated DNA damage in intestinal cancer progression, we examined the relationship between serum 8-OxodG and three key endpoints associated with cancer risk, i.e., crypt cell proliferation, metaplasia, and tumor frequency. To explore temporal trends and the distinct biological impact of radiation quality, we assessed the correlation between post-exposure time (60 and 150 days) and the ^28^Si-to-γ response ratio across all endpoints. Pearson correlation coefficients (r) were used to quantify linear relationships, and given only two time points per comparison, r values of +1.0 showed perfect positive trends. All four endpoints, including serum 8-OxodG levels, demonstrated a perfect positive correlation (r = +1.0) with increasing post-exposure time in the ^28^Si-to-γ response ratio (Figure 6). These results indicate that contrary to γ, the biological responses to ^28^Si-ion radiation intensify over time. Importantly, the strong and consistent temporal association of serum 8-OxodG with cell proliferation, metaplasia, and tumor burden underscores its potential utility as a non-invasive biomarker for predicting high-LET IR-induced tumorigenesis risk.

## 4. Discussion

While previous studies have evaluated systemic oxidative stress markers post-radiation [39,40,41], this study, using an intestinal cancer surrogate mouse model, uniquely correlates these changes with histopathological endpoints of intestinal cancer predisposition in regard to radiation quality. This study demonstrates that exposure to high-LET ^28^Si-ion radiation induces sustained oxidative DNA damage and progressive intestinal tumorigenic changes marked by enhanced epithelial cell proliferation and metaplastic transformation in *Apc*^1638N/+^ mice. Compared to low-LET γ-radiation, ^28^Si-ion exposure resulted in a time-dependent acceleration in tumor burden, increased frequency of invasive carcinomas, and sustained molecular alterations indicative of heightened carcinogenic risk. Previous studies using *Apc*^1638N/+^ and other mouse models have also demonstrated that exposure to high-LET IR causes increased intestinal tumor development compared to low-LET γ-radiation [8,42,43,44]. Our observation of a time-dependent acceleration in oxidative DNA damage accumulation accompanied with enhanced carcinogenic risk provides experimental proof that exposure to high-LET IR induces long-lasting non-targeted effects, which may amplify cancer risk over time [44,45].

The insight emerging from this study is the progressive intensification of oxidative DNA damage signatures at both the systemic and intestinal tissue levels after ^28^Si-ion exposure. Notably, γ-irradiated animals exhibited only modest elevations in 8-OxodG, indicating the greater effectiveness of high-LET radiation in inducing chronic oxidative stress, which is in concurrence to previous findings reported using in vitro and in vivo experimental models [21,46,47,48]. Chronic accumulations of oxidative DNA damage marked by 8-OxodG have established links with an increased rate of mutagenesis [49,50]. Notably, the coexistence of 8-OxodG in the intestine with enhanced crypt cell proliferation and concurrent increase in metaplasia represented precursors of malignant transformation [51,52]. Importantly, our findings support the emerging concept that high-LET IR-induced chronic oxidative stress is not an isolated effect but part of a broader spectrum of non-targeted effects (NTEs) attributed to several mechanisms involving mitochondrial dysfunction, increased NADPH oxidase, and cyclooxygenase-2 activity alongside decreased antioxidant defenses, compared to low-LET IR exposure [21,53,54]. High-LET radiation-induced oxidative stress likely synergizes with increased DNA damage, an altered DDR, and a diminished capacity for DNA repair [37,38], which could exacerbate initial damage and promote the formation of new lesions. Such synergistic interactions may drive the persistence of DNA damage and ultimately enhance the risk of mutagenesis and tumorigenesis, even in bystander or initially unaffected cells. This paradigm expands our understanding of IR-induced carcinogenesis beyond direct DNA hits, especially in the context of space radiation exposure where high-LET particles are prevalent.

**Figure 7:**
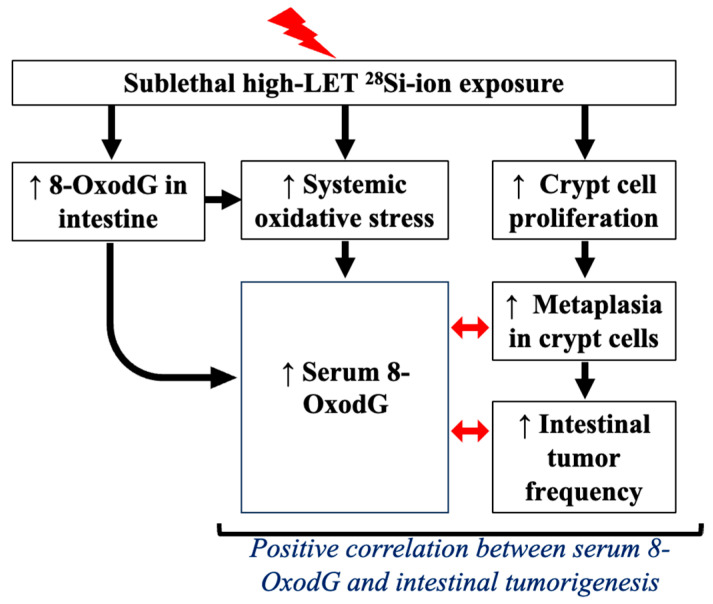
Schematic model depicting the responses to total body ^28^Si-ion exposure and its association with intestinal tumorigenesis. Exposure to ^28^Si-ions triggers persistent oxidative stress at both systemic and intestinal tissue levels. Elevated systemic (serum) 8-OxodG levels positively correlate (red arrow) with histopathological markers of intestinal tumorigenesis, including crypt cell proliferation, metaplasia, and tumor frequency. Upward arrow sign indicates upregulation after ^28^Si-ion exposure. This model supports the plausibility of serum 8-OxodG as a potential biomarker for monitoring radiation quality-dependent intestinal cancer risk.

Collectively, our study demonstrates that ^28^Si-ion radiation drives sustained oxidative damage, proliferative dysregulation, and epithelial metaplasia in the intestinal tract, leading to progressive tumorigenesis (Figure 7). These non-targeted and progressive biological effects underscore the qualitative differences between high- and low-LET radiation and reinforce the unique risks posed by space-relevant heavy ions. This study clearly demonstrates a relationship between radiation quality and cancer risk. Moreover, the elevation of serum 8-OxodG in conjunction with tissue biomarkers of proliferation and metaplasia provides a compelling rationale for its further development as a biomarker for radiation-induced carcinogenesis. Although this study was conducted using *Apc*^1638N/+^ mice, where alterations in the *Apc* gene and its encoded protein may impair the repair of oxidatively damaged DNA, similar effects have also been observed in wild-type mice and in *Apc*-independent colorectal cancer models [20,43]. This indicates that the elevation of serum 8-OxodG is not exclusively driven by *Apc* deficiency. While individual biomarkers, such as serum 8-OxodG, may correlate with tumorigenesis risk, integrating multiple complementary colorectal cancer biomarkers may enhance the precision and predictive accuracy needed to assess individual-level risk more effectively [55].

From a translational standpoint, the strong correlation between serum 8-OxodG levels and tumor outcomes highlights its potential as a biomarker for monitoring IR-induced carcinogenic risk. To mitigate this risk, promising medical countermeasure strategies include agents that (i) target sources of persistent oxidative stress and associated pro-inflammatory signaling [56], and (ii) enhance the activity or expression of key base excision repair enzymes such as 8-oxoguanine DNA glycosylase (OGG1), which specifically excises 8-OxodG lesions. Interestingly, senotherapeutic agents including metformin, fisetin, ABT-263, pterostilbene, and nicotinamide riboside have demonstrated the ability to suppress IR-induced oxidative stress and inflammatory signaling, reduce intestinal tumorigenesis in preclinical models, and promote DNA repair processes, including OGG1-mediated repair [21,57,58,59,60,61,62]. Given that persistent oxidative DNA damage is a hallmark of high-LET IR exposure-induced cancer and accelerated aging phenotypes, these interventions hold promise for reducing the risk of both cancer and non-malignant late effects during and after deep space missions. Further mechanistic studies are essential to dissect the signaling networks linking oxidative stress to high-LET radiation-induced pathologies. These insights will refine the development of targeted preventive and therapeutic approaches critical not only for astronaut health during prolonged spaceflight, but also for managing off-target effects of heavy ion-based radiotherapies on normal tissues.

## 5. Conclusions

Our findings show that exposure to ^28^Si-ions leads to increased oxidative DNA damage, cell proliferation, metaplasia, and a time-dependent rise in intestinal tumor incidence, with more carcinomas appearing at later stages. Serum 8-OxodG levels correlated with tumor burden, suggesting its potential as a predictive biomarker. Compared to γ-rays, ^28^Si-ions caused greater tumorigenesis, highlighting the radiation quality-dependent effects of high-LET exposure. These findings emphasize the heightened carcinogenic potential of high-LET radiation and identify persistent oxidative DNA damage as a critical non-targeted effect contributing to intestinal tumorigenesis, with significant implications for assessing and mitigating cancer risk in astronauts exposed to space radiation.

## Data Availability

The data presented in this study are available in this article.

## Abbreviations

The following abbreviations are used in this manuscript:

Apc	Adenomatous polyposis coli gene
ANOVA	Analysis of Variance
CRC	Colorectal cancer
cGy	Centigray
DAB	3,3′-diaminobenzidine
DDR	DNA damage response
GUCY2C	Guanylyl Cyclase C
IR	Ionizing radiation
LET	Linear energy transfer
NTEs	Non-targeted effects
OGG1	8-oxoguanine DNA glycosylase
RBE	Relative biological effectiveness
8-OxodG	8-oxo-7,8-dihydro-2′-deoxyguanosine
